# Heterogeneous detection of circulating tumor cells in patients with colorectal cancer by immunomagnetic enrichment using different EpCAM-specific antibodies

**DOI:** 10.1186/1472-6750-10-35

**Published:** 2010-04-28

**Authors:** Dalibor Antolovic, Luis Galindo, Anina Carstens, Nuh Rahbari, Markus W Büchler, Jürgen Weitz, Moritz Koch

**Affiliations:** 1 Department of Surgery, University of Heidelberg, Heidelberg, Germany

## Abstract

**Background:**

Circulating tumor cells (CTC) and disseminated tumor cells (DTC) are thought to be responsible for metastasis, so the detection of CTC may serve as individual prognostic factor in patients suffering from colorectal cancer. Therefore, a series of immunomagnetic enrichment methods for CTC have been developed using a variety of monoclonal antibodies against the Epithelial Cell Adhesion Molecule (EpCAM). However, it remains unclear whether all commercially available EpCAM antibodies show the same sensitivity and specificity. Furthermore, it remains unclear which method of sample preparation and cell extraction is most suitable for immunomagnetic enrichment and detection of CTC. In this study, we aimed to investigate whether the detection of CTC by a cytokeratin 20 reverse transcriptase-polymerase chain reaction (CK20 RT-PCR) may be influenced by the use of various Epithelial Cell Adhesion Molecule (EpCAM) antibodies for immunomagnetic isolation of CTC.

**Results:**

Using both EpCAM antibodies (mAb BerEP4 and mAb KS1/4) for immunomagnetic enrichment in blood samples of 39 patients with colorectal cancer we found heterogenous results in each patient with regard to tumor cell detection. In the tumor cell spiking experiments with whole blood samples the sensitivity of the CK 20 RT-PCR assay was higher using immunomagnetic beads coated with mAb KS1/4 compared to precoated mAb BerEP4 Dynabeads. Extraction of MNC fraction with Ficoll gradient centrifugation prior to immunomagnetic enrichment resulted in a higher sensitivity of the CK 20 RT-PCR assay.

**Conclusions:**

We concluded that isolation and detection of CTC with immunomagnetic enrichment methods is critically dependent on the used EpCAM clone. Further studies with a larger number of patients should clarify if the enrichment protocol influences the prognostic value of the tumor cell detection protocol.

## Background

Detection of circulating tumor cells (CTC) in blood and disseminated tumor cells (DTC) in the bone marrow and/or lymph nodes, which are thought to be responsible for metastases, may allow a better prediction of the individual prognosis of patients with colorectal cancer [1-3]. Recent studies of our group indicated that the molecular detection of CTC and DTC in patients with colorectal cancer (CRC) may be of prognostic value [4-7]. Furthermore, immunomagnetic enrichment strategies have been developed to improve the detection and yield of CTC and DTC [8]. A large number of monoclonal antibodies (mAb) against the Epithelial Cell Adhesion Molecule (EpCAM) which is expressed only in epithelium and malignant tumors derived from epithelia have been increasingly used to enrich and isolate CTC from blood and DTC from bone marrow samples [9,10]. However, there are no data available comparing antibodies against various EpCAM epitopes for immunomagnetic isolation of CTC with regard to their sensitivity and specificity. Therefore, it remains unclear if all anti-EpCAM antibodies are able to detect and to capture the same range of CTC and if they have the same clinical and prognostic impact. Furthermore, it is still unknown which method of sample preparation and cell extraction is most suitable for immunomagnetic enrichment and detection of CTC.

In this study, we aimed to compare two different specific antibodies against the epitope in the EGF-like domain I of EpCAM for immunomagnetic enrichment and subsequent detection of CTC in CRC patients. We used commercially available immunomagnetic beads coated with mAb BerEP4 [11] and magnetic beads coated with mAb KS1/4 [12]. Both monoclonal antibodies recognize specific epitopes of the extracellular domain of the EpCAM molecule. mAb BerEP4 recognizes two (34 kDa and 39 kDa) specific antigens, whereas mAb KS1/4 recognizes one (40 - 42 kDa) specific antigen of the extracellular domain of the EpCAM molecule [10]. Furthermore, we examined the effect of two different cell extraction protocols on subsequent immunomagnetic enrichment and detection of tumor cells in the blood.

## Results

### Specificity of the enrichment and extraction protocols

Both whole blood and MNC fractions of five healthy donors were tested regarding the specificity of cell extraction and enrichment protocols with immunomagnetic beads coated with BerEP4 and KS1/4. No CK20 signal was observed in all examined blood samples of healthy donors, demonstrating the specificity of the used assays.

### Sensitivity of the enrichment and extraction protocols

#### Whole Blood

In the tumor cell spiking experiments with whole blood samples the sensitivity of the CK20 RT-PCR assay was higher using immunomagnetic beads coated with mAb KS1/4 compared to precoated mAb BerEP4 Dynabeads. In serial dilution assays, a minimum number of 10^4 ^HT29 cells could be detected in 5 ml whole blood using the BerEP4 mAb whereas 10^3 ^HT29 cells could be detected in the same volume using the KS1/4 mAb.

#### Mononuclear cell fraction

Extraction of MNC fraction with Ficoll gradient centrifugation prior to immunomagnetic enrichment of blood samples spiked with HT29 cells resulted in a higher sensitivity of the CK20 RT-PCR assay. 10^3 ^HT29 cells spiked in 5 ml blood (200 cells/ml) could be detected after isolation of the MNC fraction using the mAb BerEP4 Dynabeads, whereas 10^2 ^HT29 cells spiked in 5 ml blood (20 cells/ml) were detected using the mAb KS1/4 coated beads.

Blood spiking experiments were repeated several times to confirm the above mentioned results.

The observed higher sensitivity of tumor cell detection after isolation of the MNC fraction prior to immunomagnetic CTC enrichment prompted us to generally use Ficoll gradient centrifugation before further immunomagnetic enrichment and detection of CTC in the blood of CRC patients.

### Detection of tumor cells in blood samples of CRC patients

Median age of included CRC patients was 63 years (range 27 - 90) with 16 females and 23 males. Eighteen of 39 (46%) patients presented with metastatic disease to the liver and were classified as UICC stage IV; 7 patients were UICC stage III, and 7 patients stage UICC II and I. Clinical data of the patients are shown in Table 1.

**Table 1:** Clinical data of our patient cohort with colorectal cancer.

Pat. No.	Age	Gender	Tumor Location	TNM	UICC	Multimodal Therapy before Surgery	Therapy
1	67	F	Metachronous liver metastasis from Colon Cancer	Tx Nx M1	IV	adjuvant	Liver resection
2	81	M	Rectum	T3 N2 M0	III	no	Anterior Rectal Resection
3	63	F	Rectum	T1 N0 M0	I	no	Anterior Rectal Resection
4	65	M	Rectum	T3 N0 M0	II	neoadjuvant	Anterior Rectal Resection
5	71	M	Colon, right	T2 N0 M0	I	no	Colectomy, right
6	60	M	Synchronous liver metastasis from Rectal Cancer	T2 N0 M1	IV	no	Anterior Rectal Resection
7	45	F	Colon, left	T4 N1 M0	III	no	Colectomy, left
8	68	F	Colon, right	T1 N0 M0	I	no	Colectomy, right
9	68	M	Metachronous liver metastasis from Rectal Cancer	Tx Nx M1	IV	neoadjuvant	Liver resection
10	78	F	Local Relapse of Rectal Cancer	T4 N0 M0	II	adjuvant	Abdominoperineal Rectal Resection and IORT*
11	62	M	Rectum	T3 N0 M0	II	no	Anterior Rectal Resection
12	53	M	Metachronous liver metastasis from Colon cancer	Tx Nx M1	IV	adjuvant	Hemihepatectomy, right
13	64	M	Synchronous liver and lung metastasis from Colon Cancer	T4 N2 M1	IV	no	palliative Colectomy, right
14	45	M	Local Relapse of Rectal Cancer	T3 N0 M0	II	adjuvant	Anterior Rectal Resection and IORT*
15	74	F	Colon, left	T1 N0 M0	I	no	Colectomy, left
16	52	M	Colon, left	T1 N0 M0	I	no	Colectomy, left
17	78	M	Colon, right	T3 N1 M0	III	no	Colectomy, right
18	72	F	Colon, right	T1 N0 M0	I	no	Colectomy, right
19	54	F	Colon, left	T4 N0 M0	II	no	Anterior Rectal Resection and IORT*
20	54	F	Metachronous liver metastasis from Colon Cancer	Tx Nx M1	IV	adjuvant	Hemihepatectomy, right
21	64	F	Metachronous liver metastasis from Rectal Cancer	Tx Nx M1	IV	no	palliative loop-Ileostomy
22	44	M	Synchronous liver metastasis from Colon Cancer	T3 N2 M1	IV	no	Anterior Rectal Resection
23	73	M	Metachronous liver metastasis from Rectal Cancer	Tx Nx M1	IV	no	palliative loop-Ileostoma
24	63	M	Metachronous liver metastasis from Colon Cancer	T3 N0 M1	IV	adjuvant	Rectosigmoid Resection
25	27	M	Colon, right	T4 N2 M0	III	no	Colectomy, right
26	68	F	Colon, right	T3 N1 M0	III	no	Colectomy, right
27	47	M	Rectum	T3 N1 M0	III	neoadjuvant	Anterior Rectal Resection and IORT*
28	68	M	Synchronous liver and bone metastasis from Colon Cancer	Tx Nx M1	IV	no	palliative Bypass Ileotransversostomy
29	76	M	Metachronous liver metastasis from Colon Cancer	Tx Nx M1	IV	adjuvant	Liver resection
30	62	M	Metachronous liver metastasis from Colon Cancer	Tx Nx M1	IV	adjuvant	Exploration, Biopsy
31	62	M	Rectum	T1 N0 M0	I	no	Anterior Rectal Resection
32	78	F	Metachronous liver metastasis from Rectal Cancer	Tx Nx M1	IV	no	Segment 2 and 3 Liver resection
33	80	M	Colon, left	T4 N0 M0	II	no	Anterior Rectal Resection
34	86	F	Rectum	T3 N0 M0	II	no	Anterior Rectal Resection
35	44	F	Local Relapse Colon Cancer, Peritonealcarcinosis	T4 N2 M1	IV	adjuvant	Laparotomy and Biopsy
36	90	F	Colon, left	T4 N1 M0	III	no	Colectomy, left
37	55	M	Metachronous liver metastasis from Rectal Cancer	Tx Nx M1	IV	adjuvant	Hemihepatectomy, right
38	56	M	Synchronous liver metastasis from Colon Cancer	T4 N1 M1	IV	adjuvant	Hemihepatectomy, left
39	63	F	Metachronous liver metastasis from Rectal Cancer	Tx Nx M1	IV	adjuvant	Hemihepatectomy, right

*Abbreviations: IORT: intraoperative radiotherapy; F: female; M: male; Pat. No.: Patients number.

**Figure 1:**
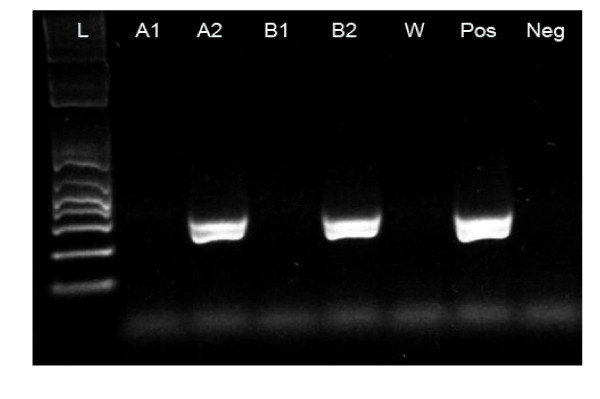
**Cytokeratin 20 RT-PCR amplification products after immunomagnetic enrichment with both Ep-CAM antibodies from blood samples of two CRC patients**. W, water negative control; Pos positive control (HT29 cells); Neg, negative control (blood of healthy person); L, ladder (molecular weight marker). Lanes A1-A2, patient ID 22 and Lanes B1-2, patient ID 23. A2 and B2 show CK20 products after CTC enrichment with KS1/4 beads whereas BerEP4 beads failed to detect CTC in the same patients (A1 and B1).

Using two different antibodies (mAb BerEP4 and mAb KS1/4) for immunomagnetic enrichment, CTC were detected in 11 of 39 (28%) patients with CRC. Among these, immunomagnetic enrichment with mAb BerEP4 beads accounted for 6 CK20 positive patients. Immunomagnetic enrichment using mAb KS1/4 beads showed 5 CK20 positive patients. Interestingly, there were no blood samples being CK 20 positive for both used antibodies (Table 2 and Figure 1).

**Table 2:** Characteristics of the CK20 positive patients and comparison of the two different antibodies used for immunomagnetic enrichment

Pat. No.	Diagnosis	TNM	UICC-Stage	BerEP4-Beads	KS 1/4-Beads
2	Rectal Cancer	T3 N2 M0	III	**positive**	negative
5	Left Colon Cancer	T2 N0 M0	I	negative	**positive**
9	Metachronous liver metastasis from Rectal Cancer	Tx Nx M1	IV	**positive**	negative
17	Right Colon Cancer	T3 N1 M0	III	**positive**	negative
22	Synchronous liver metastasis from Colon Cancer	T3 N2 M1	IV	negative	**positive**
23	Metachronous liver metastasis from Rectal Cancer	Tx Nx M1	IV	negative	**positive**
25	Right Colon Cancer	T4 N2 M0	III	**positive**	negative
28	Synchronous liver and bone metastasis from Colon Cancer	Tx Nx M1	IV	**positive**	negative
31	Rectal cancer	T1 N0 M0	I	negative	**positive**
32	Metachronous liver metastasis from Rectal Cancer	Tx Nx M1	IV	**positive**	negative
37	Metachronous liver metastasis from Rectal Cancer	Tx Nx M1	IV	negative	**positive**

CK20 PCR transcripts were detected in 3 of 18 (17%) blood samples from UICC stage IV patients after immunomagnetic enrichment with mAb KS1/4 coated beads. When mAb BerEP4 beads were used, we also observed positive CK20 products in 3 of 18 (17%) UICC stage IV patients. Among the 7 patients with UICC stage III, 3 patients showed CK20 positive samples after enrichment of CTC with BerEP4 beads only. Positive CK20 signals were observed in 2 of 7 patients with UICC stage I after enrichment with KS1/4 coated beads only (see Table 3). Furthermore, among 7 patients with UICC stage II no samples were found positive for CK20 using either BerEP4 or KS1/4 coated beads.

**Table 3:** Comparison of tumor cell detection with UICC stage of CRC patients after enrichment with mAb BerEP4 and KS14 coated beads.

UICC-Stage	NO. OF PATIENTS	POSITIVE CK20 SAMPLES	
		*BerEP4*	*KS1/4*
I	7	0	2
II	7	0	0
III	7	3	0
IV	18	3	3

## Discussion

The first phase of the metastatic process of malignant epithelial tumors consists of local tumor cell migration, followed by tumor cell dissemination in the blood, invasion and homing to secondary distant organs [13,14]. Despite recent developments of various therapeutic approaches, distant metastases represent the major cause of death in patients with colorectal cancer [14]. Theoretically, postoperative metastatic recurrence should develop from isolated tumor cells or micrometastases already existing at time of surgery or they can derive from tumor cells that are hematogenously shed into circulation during surgical procedures. Thus, a reliable detection method for CTC in a small volume of peripheral blood might be of high interest to improve staging and to more accurately predict patients' individual prognosis. To increase the specificity of tumor cell detection, immunomagnetic beads labeled with an epithelium-specific monoclonal antibody (mAb) haven been used to isolate CTC from blood [15].

To our knowledge, this is the first study reporting the use of mAb KS1/4 coated beads to isolate CTC from CRC patients. The sensitivity of the KS1/4 coated bead system was evaluated by HT29 tumor cell dilution experiments and reproducibly allowed the detection of about 10^2 ^HT29 cells spiked in 5 ml blood. In our reference experiments, mAb KS1/4 retrieved 10 fold more tumor cells than the commonly used mAb BerEP4 precoated beads (10^3 ^cells in 5 ml blood) after MNC gradient enrichment.

Our study also confirmed the study from Guo et al. which showed better results for MNC population extraction prior to immunomagnetic isolation of tumor cells than the immunomagnetic isolation of circulating tumor cells from whole blood [16]. Guo et al. combined immunomagnetic isolation followed by real-time RT-PCR to detect CTC in patients with colorectal cancer. This study showed that combining negative (CD45 depletion) and positive (CTC enrichment with BerEP4 beads) immunomagnetic selection successively yields a high amount of CTC (1 CTC/1 ml blood). As they used CEA as marker gene in their analyses, the authors did not assess the cytokeratin expression of the CTC, which is in our opinion the most widely used marker for CTC [16]. Moreover, negative enrichment with CD45 depletion of leucocytes may lead to a theoretical "loss" of CTC as they could stick together with the leucocytes-beads complexes. Denis et al. demonstrated CTC in whole blood samples in 4 of 5 patients with metastatic CRC using mAb BerEP coated magnetic beads and subsequent analysis with RT-PCR assays for CK8, CK19 and CK20 gene expression [15]. However, the previously described epithelial and tumor markers can also be expressed in normal peripheral blood leukocytes normally present in whole blood which may lead to false positive results [16-21]. Using spiking experiments with healthy donor blood Vlems et al. demonstrated that differences in sample handling and assay sensitivity influence CK20 detection in blood [22]. In the present study, isolation of CTC from CRC patients was only performed in the MNC population after Ficoll-gradient enrichment. In addition, Ficoll-gradients purge the MNC fraction from granulocytes, which constitutively express CK20 mRNA [19]. This is in accordance with our results as our nested RT-PCR retrieved consistently negative CK20 results in blood samples from healthy donors. Automated systems for immunomagnetic CTC isolation and subsequent immunocytochemical detection are nowadays available and usually rely on the principle of EpCAM based enrichment methods. Using the Cell Search system (Veridex) Cohen et al. demonstrated the prognostic value of CTC enumeration in the peripheral blood of metastatic CRC patients [23]. Future studies are needed to examine and compare the detection rates and prognostic differences between PCR based and immunocytochemical tumor cell detection methods after immunomagnetic enrichment of CTC.

BerEP4 and KS1/4 are high affinity and specific mAb frequently used to detect EpCAM positive cells. Interestingly, we noted that CK20 RT-PCR products were commonly not found in both samples when analyzing blood of the same patient after enrichment with either mAb BerEP4 or mAb KS/4. Other authors have already described heterogeneity in reactivity of EpCAM specific antibodies. Balzar et al. suggested that different conformational states of the cell surface EpCAM protein might hide some epitopes leading to subpopulations of EpCAM and thus heterogeneous affinity [10].

Successful CTC enrichment depends on the level of EpCAM expression in the target cell [24]. Furthermore, downregulation of cytokeratins in tumor derived cell lines and cytokeratin negative CTC have also been reported in patients with breast cancer [25,26]. These findings might hinder the adequate detection of CTC. Promising results for the detection of CK20 positive CTC in colorectal cancer patients were shown by Wong et al [27] by blocking the Fc region of the anti-BerEP4 antibody with a goat anti-mouse antibody during immunomagnetic enrichment. Using this refined immunomagnetic enrichment method Wong et al. could demonstrate a specific detection of colorectal CTC in vitro and they confirmed the clinical significance of their results in large series of colorectal cancer patients [27].

The dissimilar capture of CTC by BerEP4 and KS1/4 mAbs in our analysis might be explained by various expression of the EpCAM molecule among the examined patients. Nevertheless, our KS1/4 system was able to retrieve 10 fold more CTC compared to the BerEP4 system. The use of different EpCAM clones might enhance the detection rate of CTC. Additional investigations to assess the variations of EpCAM molecule expression among different patients will be of critical importance to identify a panel of suitable mAbs which could be used for efficient and reliable CTC isolation.

Our results also demonstrate that Ficoll-gradient isolation is a decisive step prior to the immunomagnetic enrichment of CTC from peripheral blood.

## Conclusions

Our study for the first time shows that isolation and detection of CTC with immunomagnetic enrichment methods is critically dependent on the used EpCAM clone. Further analysis regarding the clinical importance of heterogenous expression of the EpCAM molecule in CTC of CRC patients is urgently needed.

## Methods

### Blood samples from healthy donors and patients

For blood spiking experiments and for testing the specificity of the various extraction and enrichment protocols peripheral blood samples were drawn from the antecubital vein of 5 healthy donors and collected in EDTA-coated tubes (S-Monovette^®^, Sarstedt, Germany). To avoid epithelial cell contamination from skin puncture, the first 5 ml of peripheral blood were discarded. After collection, blood samples were immediately processed for further experiments.

Five ml blood samples were obtained after induction of general anesthesia (and before start of the operation) through a central venous line from 38 CRC patients (UICC stage I-IV) undergoing surgical therapy at the Department of Surgery, University of Heidelberg, Germany. Patients with histopathologically confirmed CRC were staged according to the classification of the UICC (6^th ^edition) [28]. The study protocol was approved by the ethics committee of the University of Heidelberg. Informed consent for blood sampling was obtained from each patient.

### Cell spiking experiments

For cell spiking experiments and the examination of different cell extraction methods, EpCAM and CK20 positive cells from the human colon cancer cell line HT29 were serially diluted in 5 ml blood samples taken from five different healthy donors. Dilutions performed were: 10^6^, 10^5^, 10^4^, 10^3^, 10^2^, 10 and 0 HT29 cells per 5 ml whole blood.

### HT29 cells

The human colon cancer cell line HT29 expressing EpCAM and Cytokeratin 20 (CK20) was purchased from American Type Cell Culture (Austria Branch). Cells were cultured in RPMI-1640 Medium with L-glutamine and HEPES (PAA Laboratories GmbH, Austria) supplemented with 100 U/ml penicillin, 100 μg/ml streptomycin (PAA Laboratories GmbH, Austria), and 10% fetal bovine serum (Biochrom AG, Germany) in plastic flasks at 37°C in a 5%CO_2 _atmosphere. Collection of cells was performed with help of Trypsin-EDTA (PAA Laboratories GmbH, Austria) and centrifugation at room temperature for 3 minutes at 300 g. Cells were then counted with a hemacytometer and viability was confirmed by Trypan blue stain.

### Mononuclear cell collection

The mononuclear cell (MNC) population was extracted according to the following protocol: 5 ml peripheral blood samples were carefully layered over a 15 ml Ficoll gradient (FicoLite-H^®^, Linaris, Germany density 1.077) and covered with 10 ml phosphate buffered saline solution (PBS; PAA Laboratories GmbH, Austria). The samples were spun in a centrifuge at 4°C for 30 minutes at 300 g without brake. Concentrated MNCs were harvested from the interface with the help of a disposable pipette. The isolated cells were washed once in PBS, spun in a centrifuge for 10 minutes at 300 g and resuspended in 1 ml PBS. The MNCs were counted with a hemacytometer and then resuspended at 10^7 ^cells/ml in PBS.

### Immunomagnetic enrichment and cell extraction protocols

The unspiked and spiked (with HT29 cells) blood samples (5 ml blood for each experiment) of healthy volunteers were processed using two different cell extraction protocols followed by immunomagnetic enrichment using the device of Dynal MPC-L (Dynal, Norway):

1. Whole blood samples underwent directly an immunomagnetic enrichment (10^7 ^beads per ml blood) using either Dynabeads Epithelial Enrich (Dynal, Oslo, Norway) coated with mAb BerEP4 or alternatively, Pan Mouse IgG beads (Dynal, Norway) coated (1 μg antibody/10^7 ^beads) with the mAb KS1/4 (BD PharMingen, Heidelberg, Germany). The samples were then placed in a roller at 4°C for rosetting to occur and after 30 minutes the tubes were placed in a magnetic device for 3 minutes; the blood supernatant was carefully removed and, while the tubes were still on the magnet, rosettes were washed three times with cold PBS.

2. The isolated MNC fraction of the blood sample was counted and resuspended with 10^7 ^beads, mAb BerEP4 Dynabeads Epithelial Enrich or alternatively, with Pan Mouse IgG beads coated with the mAb KS1/4, per 10^7 ^MNCs in 1 ml PBS. Further immunomagnetic processing was done in the same way as described above.

Finally, the rosetted cells were pelleted by centrifugation at 300 g for 10 minutes and resuspended in 100 μl PBS. Immediately, the samples were processed for RNA isolation.

In all blood samples from CRC patients isolation of the MNC fraction (prior to immunomagnetic enrichment using the two different EpCAM antibodies) was performed prior to further immunomagnetic enrichment. Immunomagnetic enrichment using the two different EpCAM antibodies was performed as described above.

### RNA extraction and nested RT-PCR

CK20 transcripts were detected after immunomagnetic enrichment of tumor cells either derived from blood samples spiked with HT29 cells or from blood samples drawn from CRC patients.

For the detection of tumor cells a CK 20 nested RT-PCR was performed as previously shown [12,13]. In brief, total RNA was extracted from the cells immobilized by the magnetic beads added to whole blood or MNC fractions. RNeasy Mini Kit (Qiagen, Hilden, Germany) was used to isolate total RNA according to the manufacturer's instructions. Each sample was eluted in 30 μl RNase-free water. For reverse transcription of the RNA the primer CK20 558.rev and the SuperScript II Kit (Invitrogen, Karlsruhe, Germany) was used following manufacturer's instructions. 2 μl aliquots of cDNA were used for the CK20 nested-PCR reaction using the Master Mix Kit from Promega (Wisconsin, USA) according to manufacturer's protocol.

For the first PCR cDNA was subjected to amplification of CK20 with primers 1.for (ATGGATTTCAGTCGCAGA) and 558.rev (ATGTAGGGTTAGGTCATCAAAG) in 35 amplification rounds performed at 93°C for 51 seconds, 60°C for 63 seconds, and 72°C for 42 seconds, with a final extension step at 72°C for 10 minutes. The nested PCR was performed with 8 μl PCR product of the first PCR with primer 139.for (TCCAACTCCAGACACACGGTGAACTATG) and 429.rev (CAGGACACACCGAGCATTTT GCAG) under amplification conditions as following: 93°C for 51 seconds and 72°C for 81 seconds in 35 amplification rounds. PCR products were analyzed by electrophoresis on 2% agarose gels. RNA quality and performance of reverse transcription of the analyzed samples was confirmed by RT-PCR amplification of GAPDH transcripts.

## Authors' contributions

DA, LG, and AC performed the experiments and contributed to clinical data collection and manuscript preparation. NR, MWB, and JW contributed to the conduction of the study and were involved in critical review and revision of the manuscript. MK designed the study and was responsible for manuscript preparation and revision. All authors read and approved the final manuscript
